# Editorial: Multidisciplinary treatment and precision medicine for acral and mucosal melanoma

**DOI:** 10.3389/fonc.2024.1429030

**Published:** 2024-05-30

**Authors:** Yu Xu, Yong Chen

**Affiliations:** Shanghai Cancer Center, Fudan University, Shanghai, China

**Keywords:** melanoma, acral melanoma, mucosal melanoma, immunotherapy, targeted therapy

Globally, more and more clinicians and scientists are recognizing that huge heterogeneity exists in the melanoma population, with different geographic regions, primary sites, and, especially, pathological subtypes. Compared to cutaneous melanoma, acral and mucosal subtypes present significantly different biological behaviors, clinical outcomes, and responses to current standard treatments, especially immunotherapy (1–4). The mechanisms behind this remain unclear. The four related articles in this special Research Topic, discussing various perspectives of multidisciplinary treatments for melanoma, truly represent this heterogeneity.

The limited clinical impact of imaging assessment on early-stage melanoma has been investigated by Papageorge et al. They found that regular CT, MRI, or PET scan did not influence clinical strategies and patient outcomes, whether they were performed before or after sentinel node biopsy. We believe it truly reflected the status of the real-world practice in the western world, where wait times for appointments for imaging assessments can be lengthy, and result in obvious increases to the medical economic burden. Meanwhile, as Chinese clinicians, we still insist that current cost-effective and efficient imaging tests, especially ultrasound, remain important to provide clinical staging information for sentinel node biopsy for T3-T4 melanoma, especially for acral and mucosal melanoma. These two more aggressive subtypes have a greater likelihood to develop into occult metastasis when being diagnosed.

The insightful finding from the article by Russo et al. suggested that, compared to primary lesions highly associated with sun-exposure pattern, melanomas from other body parts, especially at distant extremities, showed worse responses and overall outcomes when treated with immunotherapy. This aligned with the consensus that, when lacking a genetic UV-signature, a tumor often has lower tumor mutation burden and PD-L1 expression. One hypothesis is that the etiology of acral melanoma might be associated with chronic inflammatory cutaneous damage, which can lead to an inhibitory microenvironment generated by excessive immune stress or specific microbiome distribution, which can eventually trigger primary resistance to immunotherapy. Therefore, alternative treatment strategies should be modified according to deferent molecular characteristics.

The retrospective study by Minor et al., on the other hand, implied a positive finding about melanoma survival. In their single-center database on over four hundred Stage IV cases treated with immunotherapy, nearly ten percent of metastatic melanoma patients were still able to achieve a long-term survival exceeding 10 years. It is worth noting that one-third of the patients in this cohort were simply treated by biochemotherapy, including interleukin or interferon, rather than the modern checkpoint blockade. For a long time, melanoma has been regarded as an immunogenic disease, since a certain proportion of patients can experience a spontaneous remission. That actually contributes to the recovery of the host’s anti-tumor immunity. Future works should aim to discover prognostic or predictive biomarkers to identify patients with either good or poor outcomes, in order to realize individualized therapy.

The last article was a well-organized systemic review by Dugan et al., summarizing various aspects of the current clinical management of acral lentiginous melanoma, including epidemiology, clinical presentation, surgical treatments, and perioperative and late-stage medical treatment. Unfortunately, I have to point out that most of the clinical evidence cited in this review was still based on data from cutaneous melanomas. The majority of prospective phase III studies mentioned did not recruit enough patients with acral lentiginous subtype. This might be related to the different distributions of melanoma subtypes between the Caucasian and Asian populations. But it also reflected in the long-term lack of investment in the East-Asian market from overseas pharmaceutical companies. This disadvantage has been gradually addressed, with increasing high-quality cutting-edge research and clinical trials being conducted by Chinese investigators, many of which specifically focus on acral or mucosal melanoma.

In conclusion, there is still a great deal of work to do on clinical treatment and basic research on acral and mucosal melanoma. We believe several aspects should be investigated with top priority. Firstly, it is urgent to go back to basics to investigate the etiology for acral and mucosal melanoma. Targeting the fundamental cause might bring a breakthrough in therapeutic strategy. Secondly, establishing a universal and sustainable animal or organoid model holds the promise of further drug development and more effective basic research. Last but not least, neoadjuvant clinical trials with new compounds or promising combination treatments should be encouraged more in the future. Analyzing the pre- and post-treatment specimens will help to understand the response and resistance mechanism and to find valid biomarkers to efficiently categorize patients to receive more precise treatment.

We would like to extend our gratitude to all the authors, editors, and reviewers for their participation and support for our Research Topic.

## Author contributions

YX: Visualization, Validation, Supervision, Software, Resources, Project administration, Methodology, Investigation, Funding acquisition, Formal analysis, Data curation, Conceptualization, Writing – review & editing, Writing – original draft. YC: Writing – review & editing, Visualization, Validation, Supervision, Software, Resources, Project administration, Methodology, Investigation, Funding acquisition, Formal analysis, Data curation, Conceptualization.
